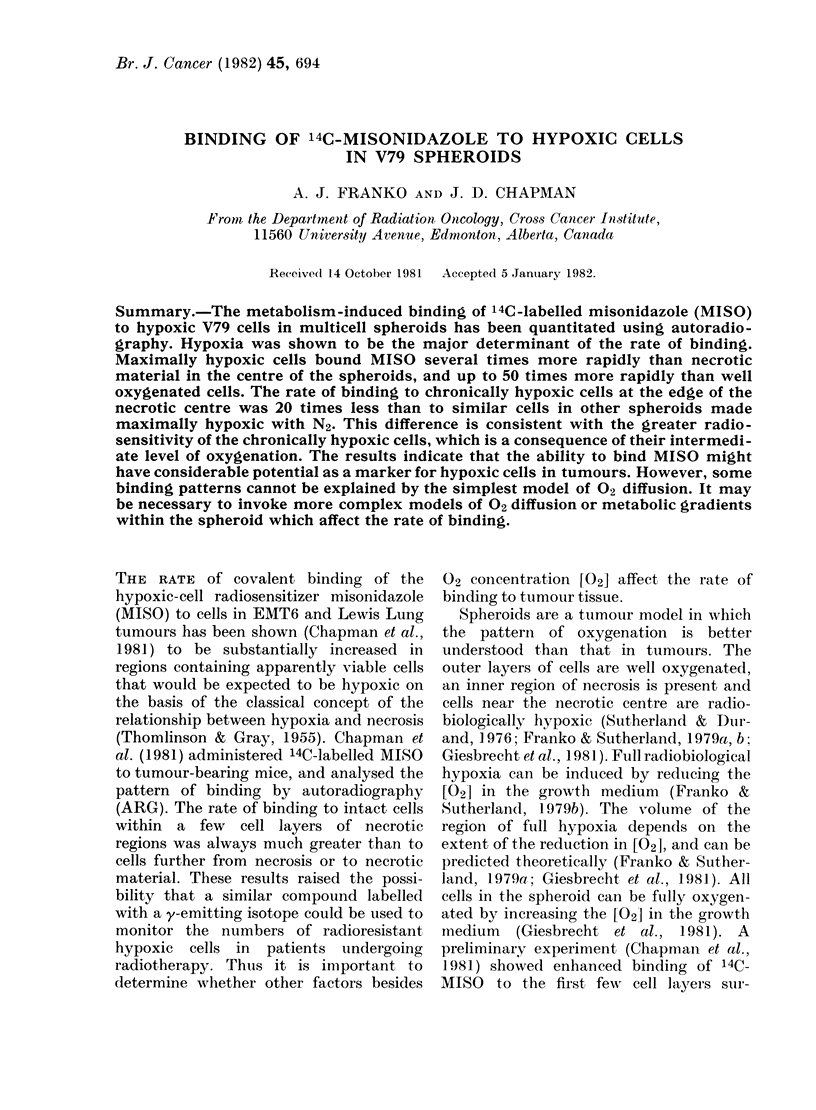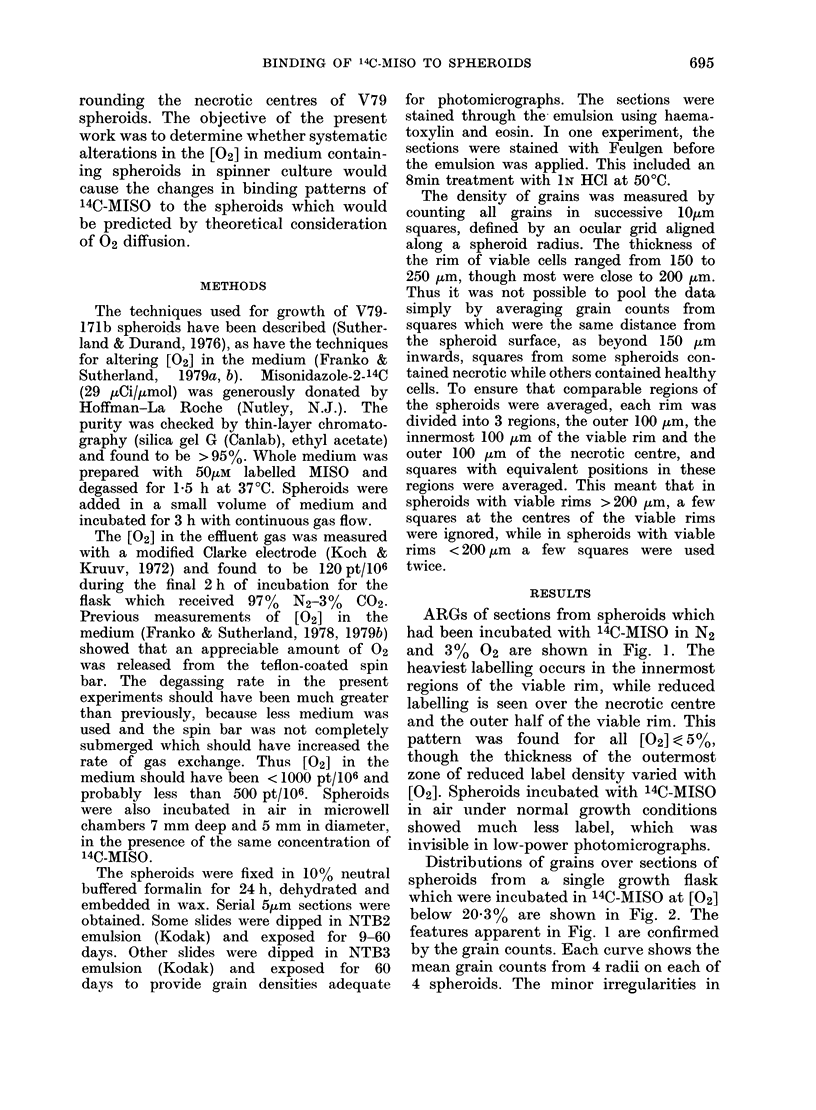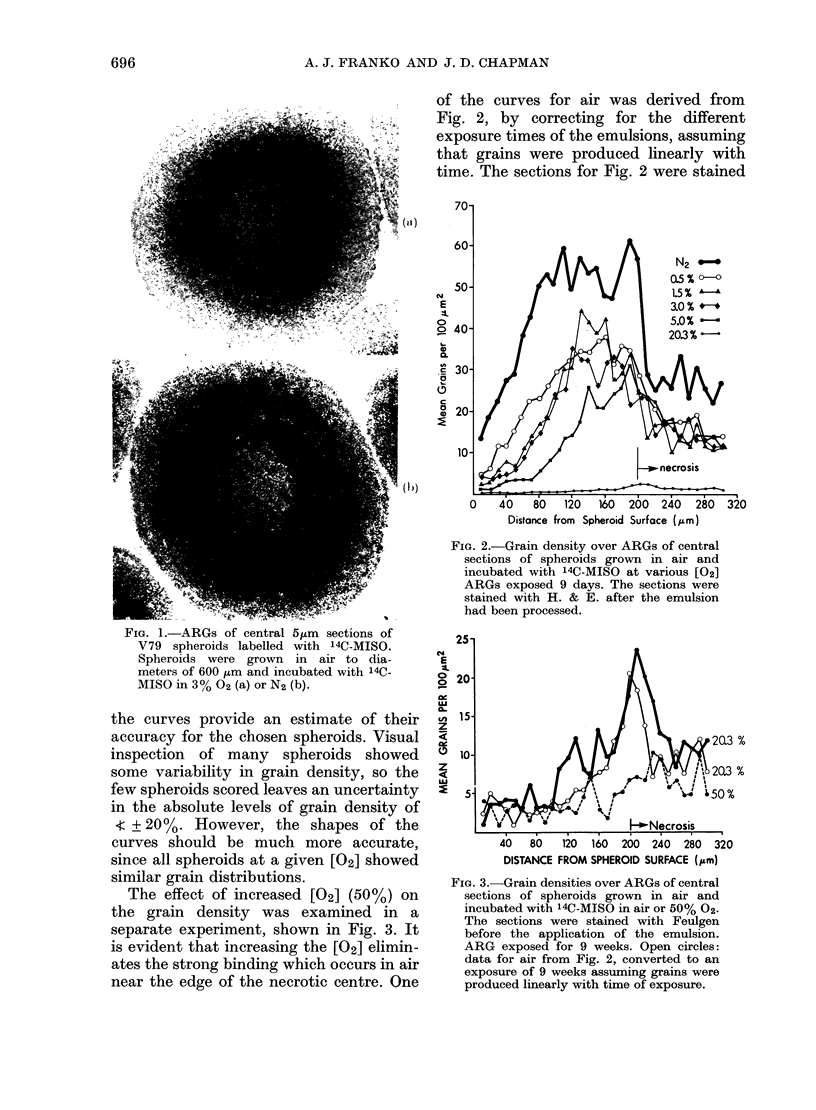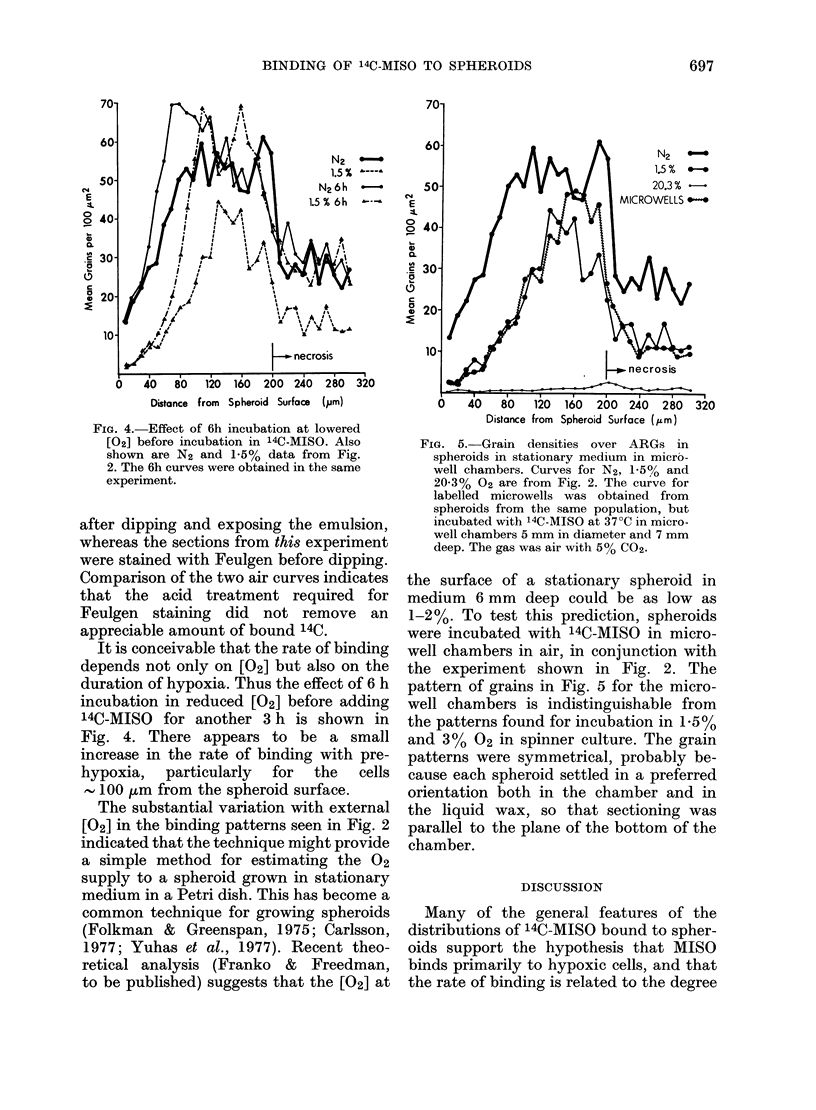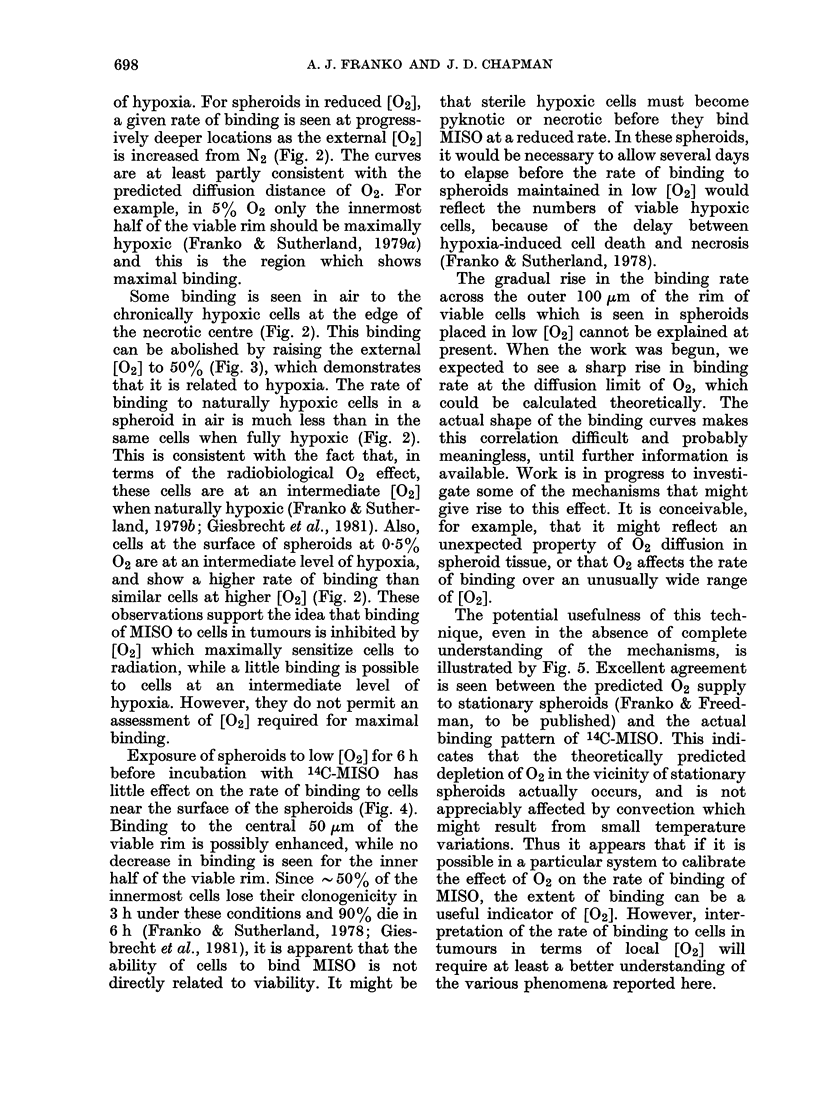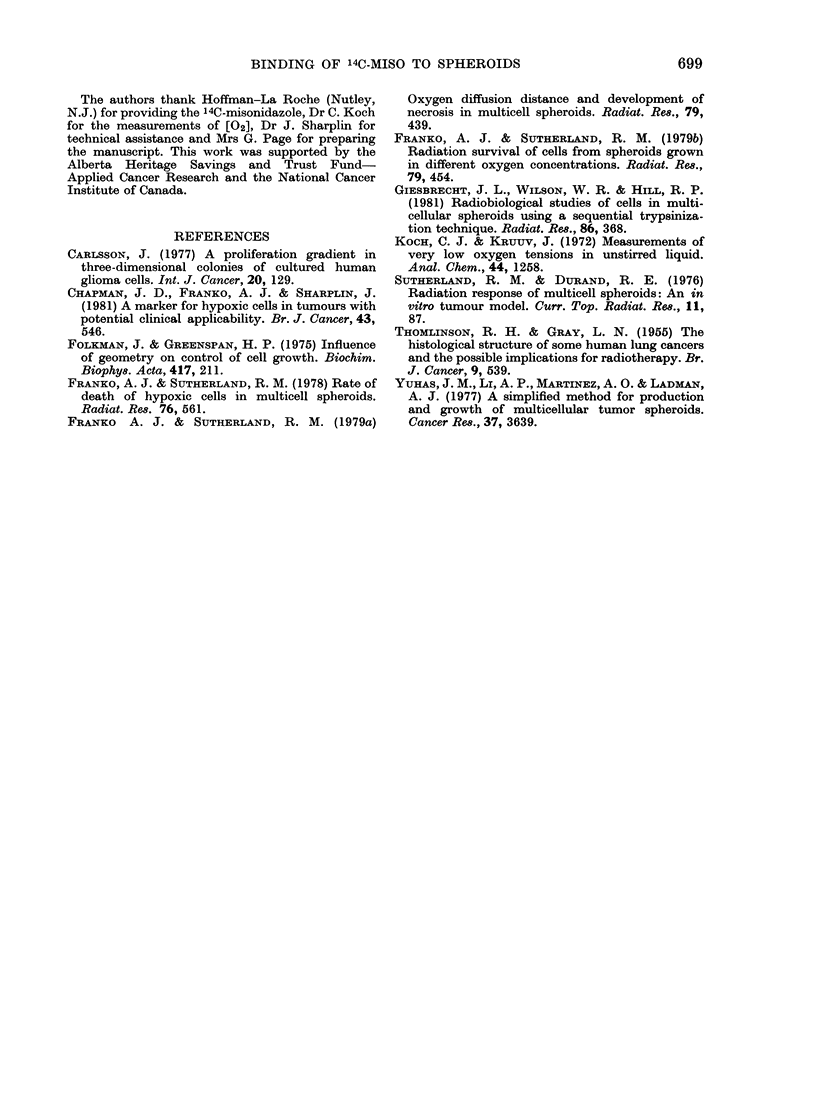# Binding of 14C-misonidazole to hypoxic cells in V79 spheroids.

**DOI:** 10.1038/bjc.1982.110

**Published:** 1982-05

**Authors:** A. J. Franko, J. D. Chapman

## Abstract

**Images:**


*Br. J. Cancer* (1982) **45**, 694

### BINDING OF $^{14}C$-MISONIDAZOLE TO HYPOXIC CELLS IN V79 SPHEROIDS

A. J. FRANKO AND J. D. CHAPMAN

*From the Department of Radiation Oncology, Cross Cancer Institute, 11560 University Avenue, Edmonton, Alberta, Canada*

Received 14 October 1981 Accepted 5 January 1982.

**Summary.—The metabolism-induced binding of $^{14}C$-labelled misonidazole (MISO) to hypoxic V79 cells in multicell spheroids has been quantitated using autoradiography. Hypoxia was shown to be the major determinant of the rate of binding. Maximally hypoxic cells bound MISO several times more rapidly than necrotic material in the centre of the spheroids, and up to 50 times more rapidly than well oxygenated cells. The rate of binding to chronically hypoxic cells at the edge of the necrotic centre was 20 times less than to similar cells in other spheroids made maximally hypoxic with $N_2$. This difference is consistent with the greater radiosensitivity of the chronically hypoxic cells, which is a consequence of their intermediate level of oxygenation. The results indicate that the ability to bind MISO might have considerable potential as a marker for hypoxic cells in tumours. However, some binding patterns cannot be explained by the simplest model of $O_2$ diffusion. It may be necessary to invoke more complex models of $O_2$ diffusion or metabolic gradients within the spheroid which affect the rate of binding.**

THE RATE of covalent binding of the hypoxic-cell radiosensitizer misonidazole (MISO) to cells in EMT6 and Lewis Lung tumours has been shown (Chapman *et al.*, 1981) to be substantially increased in regions containing apparently viable cells that would be expected to be hypoxic on the basis of the classical concept of the relationship between hypoxia and necrosis (Thomlinson & Gray, 1955). Chapman *et al.* (1981) administered $^{14}C$-labelled MISO to tumour-bearing mice, and analysed the pattern of binding by autoradiography (ARG). The rate of binding to intact cells within a few cell layers of necrotic regions was always much greater than to cells further from necrosis or to necrotic material. These results raised the possibility that a similar compound labelled with a $\gamma$-emitting isotope could be used to monitor the numbers of radioresistant hypoxic cells in patients undergoing radiotherapy. Thus it is important to determine whether other factors besides $O_2$ concentration [$O_2$] affect the rate of binding to tumour tissue.

Spheroids are a tumour model in which the pattern of oxygenation is better understood than that in tumours. The outer layers of cells are well oxygenated, an inner region of necrosis is present and cells near the necrotic centre are radiobiologically hypoxic (Sutherland & Durand, 1976; Franko & Sutherland, 1979*a*, *b*; Giesbrecht *et al.*, 1981). Full radiobiological hypoxia can be induced by reducing the [$O_2$] in the growth medium (Franko & Sutherland, 1979*b*). The volume of the region of full hypoxia depends on the extent of the reduction in [$O_2$], and can be predicted theoretically (Franko & Sutherland, 1979*a*; Giesbrecht *et al.*, 1981). All cells in the spheroid can be fully oxygenated by increasing the [$O_2$] in the growth medium (Giesbrecht *et al.*, 1981). A preliminary experiment (Chapman *et al.*, 1981) showed enhanced binding of $^{14}C$-MISO to the first few cell layers surrounding the necrotic centres of V79 spheroids. The objective of the present work was to determine whether systematic alterations in the $[O_2]$ in medium containing spheroids in spinner culture would cause the changes in binding patterns of $^{14}C$-MISO to the spheroids which would be predicted by theoretical consideration of $O_2$ diffusion.

### METHODS

The techniques used for growth of V79-171b spheroids have been described (Sutherland & Durand, 1976), as have the techniques for altering $[O_2]$ in the medium (Franko & Sutherland, 1979*a*, *b*). Misonidazole-2-$^{14}C$ (29 $\mu$Ci/$\mu$mol) was generously donated by Hoffman–La Roche (Nutley, N.J.). The purity was checked by thin-layer chromatography (silica gel G (Canlab), ethyl acetate) and found to be $>95\%$. Whole medium was prepared with 50$\mu$M labelled MISO and degassed for 1·5 h at 37°C. Spheroids were added in a small volume of medium and incubated for 3 h with continuous gas flow.

The $[O_2]$ in the effluent gas was measured with a modified Clarke electrode (Koch & Kruuv, 1972) and found to be 120 pt/$10^6$ during the final 2 h of incubation for the flask which received 97% $N_2$–3% $CO_2$. Previous measurements of $[O_2]$ in the medium (Franko & Sutherland, 1978, 1979*b*) showed that an appreciable amount of $O_2$ was released from the teflon-coated spin bar. The degassing rate in the present experiments should have been much greater than previously, because less medium was used and the spin bar was not completely submerged which should have increased the rate of gas exchange. Thus $[O_2]$ in the medium should have been $<1000$ pt/$10^6$ and probably less than 500 pt/$10^6$. Spheroids were also incubated in air in microwell chambers 7 mm deep and 5 mm in diameter, in the presence of the same concentration of $^{14}C$-MISO.

The spheroids were fixed in 10% neutral buffered formalin for 24 h, dehydrated and embedded in wax. Serial 5$\mu$m sections were obtained. Some slides were dipped in NTB2 emulsion (Kodak) and exposed for 9–60 days. Other slides were dipped in NTB3 emulsion (Kodak) and exposed for 60 days to provide grain densities adequate for photomicrographs. The sections were stained through the emulsion using haematoxylin and eosin. In one experiment, the sections were stained with Feulgen before the emulsion was applied. This included an 8min treatment with 1N HCl at 50°C.

The density of grains was measured by counting all grains in successive 10$\mu$m squares, defined by an ocular grid aligned along a spheroid radius. The thickness of the rim of viable cells ranged from 150 to 250 $\mu$m, though most were close to 200 $\mu$m. Thus it was not possible to pool the data simply by averaging grain counts from squares which were the same distance from the spheroid surface, as beyond 150 $\mu$m inwards, squares from some spheroids contained necrotic while others contained healthy cells. To ensure that comparable regions of the spheroids were averaged, each rim was divided into 3 regions, the outer 100 $\mu$m, the innermost 100 $\mu$m of the viable rim and the outer 100 $\mu$m of the necrotic centre, and squares with equivalent positions in these regions were averaged. This meant that in spheroids with viable rims $>200$ $\mu$m, a few squares at the centres of the viable rims were ignored, while in spheroids with viable rims $<200$ $\mu$m a few squares were used twice.

### RESULTS

ARGs of sections from spheroids which had been incubated with $^{14}C$-MISO in $N_2$ and 3% $O_2$ are shown in Fig. 1. The heaviest labelling occurs in the innermost regions of the viable rim, while reduced labelling is seen over the necrotic centre and the outer half of the viable rim. This pattern was found for all $[O_2] \leqslant 5\%$, though the thickness of the outermost zone of reduced label density varied with $[O_2]$. Spheroids incubated with $^{14}C$-MISO in air under normal growth conditions showed much less label, which was invisible in low-power photomicrographs.

Distributions of grains over sections of spheroids from a single growth flask which were incubated in $^{14}C$-MISO at $[O_2]$ below 20·3% are shown in Fig. 2. The features apparent in Fig. 1 are confirmed by the grain counts. Each curve shows the mean grain counts from 4 radii on each of 4 spheroids. The minor irregularities in

FIG. 1.—ARGs of central 5$\mu$m sections of V79 spheroids labelled with $^{14}$C-MISO. Spheroids were grown in air to diameters of 600 $\mu$m and incubated with $^{14}$C-MISO in 3% $O_2$ (a) or $N_2$ (b).

the curves provide an estimate of their accuracy for the chosen spheroids. Visual inspection of many spheroids showed some variability in grain density, so the few spheroids scored leaves an uncertainty in the absolute levels of grain density of $\nless \pm 20\%$. However, the shapes of the curves should be much more accurate, since all spheroids at a given [$O_2$] showed similar grain distributions.

The effect of increased [$O_2$] (50%) on the grain density was examined in a separate experiment, shown in Fig. 3. It is evident that increasing the [$O_2$] eliminates the strong binding which occurs in air near the edge of the necrotic centre. One of the curves for air was derived from Fig. 2, by correcting for the different exposure times of the emulsions, assuming that grains were produced linearly with time. The sections for Fig. 2 were stained

FIG. 2.—Grain density over ARGs of central sections of spheroids grown in air and incubated with $^{14}$C-MISO at various [$O_2$] ARGs exposed 9 days. The sections were stained with H. & E. after the emulsion had been processed.

FIG. 3.—Grain densities over ARGs of central sections of spheroids grown in air and incubated with $^{14}$C-MISO in air or 50% $O_2$. The sections were stained with Feulgen before the application of the emulsion. ARG exposed for 9 weeks. Open circles: data for air from Fig. 2, converted to an exposure of 9 weeks assuming grains were produced linearly with time of exposure.

FIG. 4.—Effect of 6h incubation at lowered [$O_2$] before incubation in $^{14}$C-MISO. Also shown are $N_2$ and 1·5% data from Fig. 2. The 6h curves were obtained in the same experiment.

FIG. 5.—Grain densities over ARGs in spheroids in stationary medium in microwell chambers. Curves for $N_2$, 1·5% and 20·3% $O_2$ are from Fig. 2. The curve for labelled microwells was obtained from spheroids from the same population, but incubated with $^{14}$C-MISO at 37°C in microwell chambers 5 mm in diameter and 7 mm deep. The gas was air with 5% $CO_2$.

after dipping and exposing the emulsion, whereas the sections from *this* experiment were stained with Feulgen before dipping. Comparison of the two air curves indicates that the acid treatment required for Feulgen staining did not remove an appreciable amount of bound $^{14}$C.

It is conceivable that the rate of binding depends not only on [$O_2$] but also on the duration of hypoxia. Thus the effect of 6 h incubation in reduced [$O_2$] before adding $^{14}$C-MISO for another 3 h is shown in Fig. 4. There appears to be a small increase in the rate of binding with prehypoxia, particularly for the cells ~ 100 μm from the spheroid surface.

The substantial variation with external [$O_2$] in the binding patterns seen in Fig. 2 indicated that the technique might provide a simple method for estimating the $O_2$ supply to a spheroid grown in stationary medium in a Petri dish. This has become a common technique for growing spheroids (Folkman & Greenspan, 1975; Carlsson, 1977; Yuhas *et al.*, 1977). Recent theoretical analysis (Franko & Freedman, to be published) suggests that the [$O_2$] at the surface of a stationary spheroid in medium 6 mm deep could be as low as 1–2%. To test this prediction, spheroids were incubated with $^{14}$C-MISO in microwell chambers in air, in conjunction with the experiment shown in Fig. 2. The pattern of grains in Fig. 5 for the microwell chambers is indistinguishable from the patterns found for incubation in 1·5% and 3% $O_2$ in spinner culture. The grain patterns were symmetrical, probably because each spheroid settled in a preferred orientation both in the chamber and in the liquid wax, so that sectioning was parallel to the plane of the bottom of the chamber.

### DISCUSSION

Many of the general features of the distributions of $^{14}$C-MISO bound to spheroids support the hypothesis that MISO binds primarily to hypoxic cells, and that the rate of binding is related to the degree of hypoxia. For spheroids in reduced [$O_2$], a given rate of binding is seen at progressively deeper locations as the external [$O_2$] is increased from $N_2$ (Fig. 2). The curves are at least partly consistent with the predicted diffusion distance of $O_2$. For example, in 5% $O_2$ only the innermost half of the viable rim should be maximally hypoxic (Franko & Sutherland, 1979*a*) and this is the region which shows maximal binding.

Some binding is seen in air to the chronically hypoxic cells at the edge of the necrotic centre (Fig. 2). This binding can be abolished by raising the external [$O_2$] to 50% (Fig. 3), which demonstrates that it is related to hypoxia. The rate of binding to naturally hypoxic cells in a spheroid in air is much less than in the same cells when fully hypoxic (Fig. 2). This is consistent with the fact that, in terms of the radiobiological $O_2$ effect, these cells are at an intermediate [$O_2$] when naturally hypoxic (Franko & Sutherland, 1979*b*; Giesbrecht *et al.*, 1981). Also, cells at the surface of spheroids at 0·5% $O_2$ are at an intermediate level of hypoxia, and show a higher rate of binding than similar cells at higher [$O_2$] (Fig. 2). These observations support the idea that binding of MISO to cells in tumours is inhibited by [$O_2$] which maximally sensitize cells to radiation, while a little binding is possible to cells at an intermediate level of hypoxia. However, they do not permit an assessment of [$O_2$] required for maximal binding.

Exposure of spheroids to low [$O_2$] for 6 h before incubation with $^{14}$C-MISO has little effect on the rate of binding to cells near the surface of the spheroids (Fig. 4). Binding to the central 50 $\mu$m of the viable rim is possibly enhanced, while no decrease in binding is seen for the inner half of the viable rim. Since $\sim$50% of the innermost cells lose their clonogenicity in 3 h under these conditions and 90% die in 6 h (Franko & Sutherland, 1978; Giesbrecht *et al.*, 1981), it is apparent that the ability of cells to bind MISO is not directly related to viability. It might be that sterile hypoxic cells must become pyknotic or necrotic before they bind MISO at a reduced rate. In these spheroids, it would be necessary to allow several days to elapse before the rate of binding to spheroids maintained in low [$O_2$] would reflect the numbers of viable hypoxic cells, because of the delay between hypoxia-induced cell death and necrosis (Franko & Sutherland, 1978).

The gradual rise in the binding rate across the outer 100 $\mu$m of the rim of viable cells which is seen in spheroids placed in low [$O_2$] cannot be explained at present. When the work was begun, we expected to see a sharp rise in binding rate at the diffusion limit of $O_2$, which could be calculated theoretically. The actual shape of the binding curves makes this correlation difficult and probably meaningless, until further information is available. Work is in progress to investigate some of the mechanisms that might give rise to this effect. It is conceivable, for example, that it might reflect an unexpected property of $O_2$ diffusion in spheroid tissue, or that $O_2$ affects the rate of binding over an unusually wide range of [$O_2$].

The potential usefulness of this technique, even in the absence of complete understanding of the mechanisms, is illustrated by Fig. 5. Excellent agreement is seen between the predicted $O_2$ supply to stationary spheroids (Franko & Freedman, to be published) and the actual binding pattern of $^{14}$C-MISO. This indicates that the theoretically predicted depletion of $O_2$ in the vicinity of stationary spheroids actually occurs, and is not appreciably affected by convection which might result from small temperature variations. Thus it appears that if it is possible in a particular system to calibrate the effect of $O_2$ on the rate of binding of MISO, the extent of binding can be a useful indicator of [$O_2$]. However, interpretation of the rate of binding to cells in tumours in terms of local [$O_2$] will require at least a better understanding of the various phenomena reported here.

The authors thank Hoffman-La Roche (Nutley, N.J.) for providing the $^{14}C$-misonidazole, Dr C. Koch for the measurements of $[O_2]$, Dr J. Sharplin for technical assistance and Mrs G. Page for preparing the manuscript. This work was supported by the Alberta Heritage Savings and Trust Fund—Applied Cancer Research and the National Cancer Institute of Canada.

### REFERENCES

CARLSSON, J. (1977) A proliferation gradient in three-dimensional colonies of cultured human glioma cells. *Int. J. Cancer*, **20**, 129.

CHAPMAN, J. D., FRANKO, A. J. & SHARPLIN, J. (1981) A marker for hypoxic cells in tumours with potential clinical applicability. *Br. J. Cancer*, **43**, 546.

FOLKMAN, J. & GREENSPAN, H. P. (1975) Influence of geometry on control of cell growth. *Biochim. Biophys. Acta*, **417**, 211.

FRANKO, A. J. & SUTHERLAND, R. M. (1978) Rate of death of hypoxic cells in multicell spheroids. *Radiat. Res.* **76**, 561.

FRANKO A. J. & SUTHERLAND, R. M. (1979*a*) Oxygen diffusion distance and development of necrosis in multicell spheroids. *Radiat. Res.*, **79**, 439.

FRANKO, A. J. & SUTHERLAND, R. M. (1979*b*) Radiation survival of cells from spheroids grown in different oxygen concentrations. *Radiat. Res.*, **79**, 454.

GIESBRECHT, J. L., WILSON, W. R. & HILL, R. P. (1981) Radiobiological studies of cells in multicellular spheroids using a sequential trypsinization technique. *Radiat. Res.*, **86**, 368.

KOCH, C. J. & KRUUV, J. (1972) Measurements of very low oxygen tensions in unstirred liquid. *Anal. Chem.*, **44**, 1258.

SUTHERLAND, R. M. & DURAND, R. E. (1976) Radiation response of multicell spheroids: An *in vitro* tumour model. *Curr. Top. Radiat. Res.*, **11**, 87.

THOMLINSON, R. H. & GRAY, L. N. (1955) The histological structure of some human lung cancers and the possible implications for radiotherapy. *Br. J. Cancer*, **9**, 539.

YUHAS, J. M., LI, A. P., MARTINEZ, A. O. & LADMAN, A. J. (1977) A simplified method for production and growth of multicellular tumor spheroids. *Cancer Res.*, **37**, 3639.